# Pre-migratory flights in migrant songbirds: the ecological and evolutionary importance of understudied exploratory movements

**DOI:** 10.1186/s40462-023-00440-y

**Published:** 2023-12-19

**Authors:** Zephyr Züst, Andrey Mukhin, Philip D. Taylor, Heiko Schmaljohann

**Affiliations:** 1 Institute for Biology and Environmental Sciences (IBU), Carl von Ossietzky Universität Oldenburg, Oldenburg, Germany; 2 Zoological Institute Russian Academy of Science, Biological Station Rybachy, Kaliningrad Oblast, Russia; 3 Department of Biology, Acadia University, Wolfville, NS Canada

## Abstract

Across the animal kingdom, from honeybees to cranes to beavers, exploratory movements to exploit resources, scout prospective territories, or otherwise gain valuable experiences and information that promote fitness have been documented. For example, exploratory movements to investigate potential dispersal targets have been observed in roe deer, Northern cardinals, and tigers alike. However, despite how widespread these movements are, a cohesive definition of exploratory movements has been lacking. We first provide a clear definition of exploratory movements, and use one particular group—migratory songbirds—to catalogue exploratory movements across the annual cycle. The exceptional mobility of migratory songbirds results in exploratory movements not only at a local scale, but also on a regional scale, both in and out of the breeding season. We review the extent to which these movements are made within this group, paying particular attention to how such movements confer fitness benefits, as by securing high-quality territories, prospecting for extra-pair paternity, or even exploiting ephemeral resources. We then zoom in one step further to a particular exploratory movement that has been, to date, almost completely overlooked within this group: that of pre-migratory flights. These flights, which occur during the transitional period between the stationary breeding period and the onset of migration, occur at night and may not be made by all individuals in a population—reasons why these flights have been heretofore critically understudied. We provide the first definition for this behaviour, summarise the current knowledge of this cryptic movement, and hypothesise what evolutionary/ecological advantages conducting it may confer to the individuals that undertake it. As these flights provide experience to the individuals that undertake them, we expect that birds that make pre-migratory flights are better equipped to survive migration (direct fitness benefits) and, due to orientation/navigation abilities, may also reach preferred territories on breeding and wintering grounds faster (indirect fitness benefits). We hope to encourage ecologists to consider such hidden movements in their research concepts and to enhance the framework of movement ecology by this behaviour due to its presumed high biological importance to the annual cycle of birds.

## Background

As animals move through an unfamiliar environment, they inherently expose themselves to unknown threats. The costs of these exploratory movements are outweighed by various gains, such as access to limited resources (food, mates, habitat) or encountering characteristic landscape features that may aid navigation. Thus, despite possible risks associated with exploration of unfamiliar areas, the benefits at least balance the costs.

Notably, exploratory movements have been documented across nearly all vertebrate groups and insects (for insect reviews, see [2–4]). Age, experience, and life stage play a role in when, and to what extent, exploratory movements are made. For example, in mammals, exploratory movements preceding dispersal are widely observed [5–13], and appear to be a precursor to successful dispersal [14]. Excursions in apparent pursuit of breeding opportunities in mammals have also been recorded [15–19]. Among amphibians, the study of exploration behaviour has been “deeply neglected” [20], although invasive cane toads *Rhinella marina* at the front edge of their expanding range show higher levels of exploratory behaviour than those from the core of the range [21], suggesting a willingness to move in unfamiliar landscapes, likely facilitating their invasion [22]. When fewer females were present within their territories, male Caucasian rock lizards *Darevskia saxicola* made more exploratory movements beyond their territory borders [23]. Even freshwater fish have been documented making periodic exploratory movements outside of their home pools to assess alternative home sites [24].

Although they may not be universally conducted within the group or even within a given species, exploratory movements in birds have been especially well-documented. To find ephemeral food resources or to locate better feeding conditions when food availability on-territory is scarce, birds make exploratory movements off-territory [25–28]. Juveniles make exploratory movements prior to emancipation or dispersal [29–31], and both subadults and adults make regional exploratory movements to prospect future breeding sites [32–34]. Broadly, exploratory movements have been observed across avian taxa, including cranes [35], prairie-chickens *Tympanuchus *spp*.* [36, 37], and songbirds [38–40].

These examples demonstrate the extent to which exploratory movements can occur across the animal kingdom, which underlines this behaviour as a fundamental trait for ecology and evolution. However, despite the clear abundance of cases in which exploratory movements have been observed, we found little consensus on how, exactly, an exploratory movement is defined. (A subcategory is the “exploratory foray,” though this is typically limited to individuals with a defined territory, as summarised in [41]). Often, an exploratory movement is termed as such, without any explanation of what criteria were used for this designation. Exploratory movement has been defined as:

(i) movement outside an individual’s home range [14], wherein individuals return to or near to that home range [42–45],
(ii) movement synonymous with prospecting, in which individuals assess habitat that they may later use as breeding territories [46, 47],and
(iii) movement that serves as a prelude to dispersal [8, 48, 49], though sometimes, the term “exploratory movement” is used synonymously with “dispersal movement” [50].

These definitions are insufficient to capture all movements that are exploratory in nature, especially as we think exploratory movements may occur during life stages even when an individual is not defending a territory, or may not serve purely to gain one’s own territory, as for individuals on migration (e.g., [51]).

Our first objective was to provide a general definition of exploratory movements. In order to be able to characterise, research, quantify, and compare exploratory movements between studies, species, populations and individuals, a cohesive definition is required. Applying this definition, we review the ecological and evolutionary importance of exploratory movements within the annual cycle in order to highlight the biological significance of such movements. For this, we focus on migratory songbirds as model organisms. In this highly motile group, exploratory movements have been documented throughout the annual cycle, making it an ideal system in which to investigate the purpose, drivers, and potential benefits of exploratory movements, especially as they may refer to movements beyond a year-round or breeding range. We then evaluate, characterise, and highlight the understudied transitional period between breeding and autumn migration [52, 53]. During this transition phase, rarely recorded exploratory movements—so-called “pre-migratory flights”—occur [54, 55]. We review the current knowledge on these flights, highlight their ecological and evolutionary importance, and postulate their significant immediate and delayed fitness benefits. Our review aims to promote research on this particular exploratory movement and stimulate scientific discussion about it within the full annual cycle perspective.

## Definition of exploratory movements

We generally define an exploratory movement as a movement with a primary purpose to exploit nearby resources and/or gain information or experience beyond an individual’s current stationary phase, such as natal/breeding area, wintering ground, or stopover site, to increase immediate (e.g., survival, extra-pair copulation) or delayed (e.g., information about favourable nest sites for future breeding season, etc.) fitness benefits. Such movement does not necessarily have to be directed with the primary purpose of relocating, but may happen in any direction and over different time periods. Such a movement may terminate at or near the stationary phase from which it was initiated (e.g., during the breeding season, on an extended stopover), or alternatively may terminate elsewhere if conducted during a transient period (e.g., dispersal), during the transition from a transient to a stationary period (e.g., arrival at breeding grounds, arrival at a stopover site), or vice versa.

## Ecological and evolutionary importance of exploratory movements within the annual cycle of migratory songbirds

**Fig. 1:**
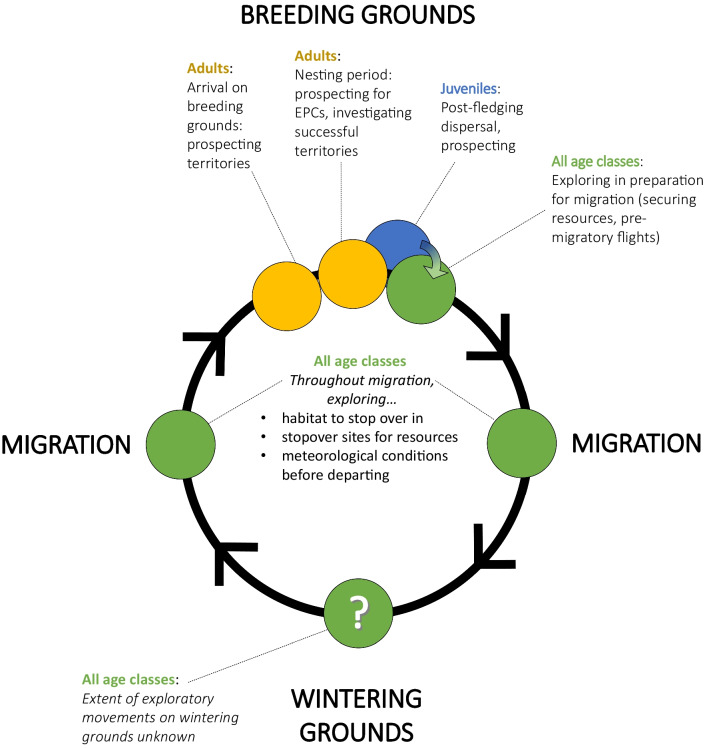
Exploratory movements have been observed in migratory songbirds across the annual cycle. These movement types are found in *all age classes*. For example, on the breeding grounds, a bird may prospect for extra-pair copulations (EPCs) or prospect other territories to evaluate how successful they have been, while on migration, a bird may explore a stopover site to locate food

We expect that exploratory movements occur both in juveniles and in adults throughout the annual cycle. Here we focus on migratory songbirds to capture the ecological and evolutionary importance of such movements. Although exploratory movements often occur unnoticed and thus are broadly understudied, examples of such exploratory movements appear to be reported more often among migratory birds compared to other groups. Within this group, exploratory movements have been observed both in territorial and non-territorial contexts across the annual cycle (Fig. 1). However, in contrast to other taxa (such as parrots and non-migratory songbirds), to the best of our knowledge, there are very few studies that focus specifically on exploratory movements for food acquisition within migratory songbirds. We speculate that such movements occur throughout the annual cycle, and surmise that due to their migratory nature, migratory birds may even be able to find and exploit ephemeral food resources faster than their non-migratory counterparts (e.g., [56]). Without further studies to support this type of exploratory movement, we focus predominantly on exploratory movements in the context of reproduction and territory acquisition below.

### Exploratory movements on the breeding grounds (adults)

Upon arrival on their breeding grounds, individuals decide where to breed. Experienced birds may occupy a territory successfully held in previous years [57, 58] or shift territories based on prior knowledge of higher quality areas [59] to maintain or increase reproductive success. They may investigate hetero- and conspecifics’ choice of territory and nest site to inform their own decisions about where and how much to invest in a breeding decision [60–64].

One reliable information source on where to settle are social cues of conspecifics, e.g., songs, which indicate favourable breeding habitats. Playback experiments that simulate this type of social information were used by both hatch year and after hatch year birds [65–67]. It was even shown that yellow warblers *Setophaga petechia* distinguish between “paired” and “unpaired” song types: more birds settled at “paired” plots, using the social cue of pair status as a proxy for habitat quality [68]. This non-random distribution strongly suggests that exploratory movements through potential breeding habitats inform settlement decisions. This was further demonstrated in nightingales *Luscinia megarhynchos* [69]. Males were translocated to a novel habitat and hence lacked any knowledge of prior breeding success. These functionally “naïve” breeders then prospected previously established male territories before establishing their own [69]. Comparable exploratory movements were observed in females of different species, similarly exploring multiple male territories before settling [70, 71], and preferentially choosing the highest-quality male [72] and/or territory [73].

As the breeding season proceeds, males and females explore other territories to engage in extra-pair copulations, often covering substantial distances (e.g., [74–80]). Notably, male hooded warblers *Setophaga citrina* move up to 2.5 km between forest fragments [81], and eastern kingbirds *Tyrannus tyrannus* [82] travel up to 15 km to secure extra-pair copulations. Male black-throated blue warblers *Setophaga caerulescens* that breed in food-scarce territories seek extra-pair copulations far beyond their immediate spatial neighbourhood, where feeding conditions might be better and may lead to higher offspring survival [83]. Further extraterritorial exploratory movements throughout the breeding season, e.g., within-season territory shifts to higher-quality areas, and female assessment of neighbouring males before the advent of their fertile period, are reviewed in Whitaker and Warkentin [84].

Over the course of the breeding season, increasingly more individuals fail to secure a mate or experience nest failure. In these cases, unsuccessful or unpaired individuals may make exploratory forays to gain information about conspecifics’ nesting success, or may explore available habitat to attempt to secure a breeding opportunity elsewhere. For example, subadult female tree swallows adopt a floater strategy and make exploratory movements in active territories, which enable them to quickly identify and exploit an available nest site [85]. Male ortolan buntings *Emberiza hortulana* that fail to secure a mate shortly after arrival on their breeding grounds continuously shift to new territories, sometimes moving more than 40 km between patches [86]. Moreover, birds may make exploratory movements to identify potential territories for future breeding attempts, gleaning social information about where conspecifics have produced nestlings and/or fledges as indicators of territory quality [87], sometimes more than 30 km away from the own territories [88]. A spectacular example was found in Kirtland’s warblers *Setophaga kirtlandii*, in which unpaired males made nocturnal flights to prospect settled territories up to 77 km away, timing their movements to match with the nestling and fledging stages of successful breeders [89]. Red-backed shrikes *Lanius collurio* also carried out movements up to 1 h around their summer territories/breeding areas, ostensibly following breeding failure [90], possibly to do similar prospecting.

Likely due to difficulty in tracking highly mobile individuals or following their movements throughout the course of an entire season or beyond, most studies document only one type of exploratory movement per species. However, multiple different exploratory movements have been characterised within a single species [91], and is indeed what we expect to observe within migratory songbirds.

In sum, exploratory movements of adults on breeding grounds serve to maximise their fitness by encountering the highest-quality mates and territories, extra-pair partners, and/or by gaining information to determine where to settle. Birds that arrive first to the breeding grounds explore available habitat and select territories with the best amenities [92], and these early-arriving birds fledge more young on their territories compared to later-arriving birds [93]. However, both early- and late-arriving birds may still take advantage of exploring other territories to secure extra-pair paternity [94]. Failed breeders that prospect successful territories have also been shown to return earlier and have higher probabilities of settling on a high-quality territory the subsequent year [95], thereby somewhat offsetting their unsuccessful attempts.

### Exploratory movements during migration

During migration, most birds encounter novel environments. This is especially true for first year long-distance migrants, which often cross completely different ecosystems from the natal sites in which they hatched. Birds may need to adjust to unfamiliar conditions or seek the most favourable site within the novel environment, so unsurprisingly, exploratory movements play an important role during the various stages of migration. After arriving at a stopover site, migrants may leave shortly afterwards in any direction to find a more suitable habitat for the purposes of stopover within the “stopover landscape”, [96–99], see also [100]. Individuals may make exploratory flights to gain familiarity within a patch, or may move continuously in an exploratory manner throughout the stopover [101, 102]. Northern wheatears *Oenanthe oenanthe* conduct exploratory flights on a stopover site prior to committing to departing, presumably to assess suitability of meteorological conditions aloft for the next migratory bout [51]. These exploratory movements have direct consequences for birds’ fitness. Should a bird fail to find sufficient resources on stopover to fuel, it may starve. If it takes too long to accumulate energy, it may arrive at its breeding or wintering grounds too late to compete for a high-quality territory, reducing its indirect fitness. Should it depart in poor conditions, it may require more time to complete its migration [103], further reducing its probability of settling on a high-quality territory. As such, exploratory movements on stopover that serve to find favourable resources [102] or assess meteorological conditions may have consequences both for an individuals’ immediate fitness (i.e. survival), but also its reproductive potential.

### Exploratory movements on the wintering grounds

Whether and to what extent exploratory movements are made on the wintering grounds is currently unknown. Nevertheless, given that exploratory movements occur throughout the rest of migratory songbirds’ annual cycle, we think it plausible that birds also perform such movements on wintering grounds, for example to locate patchily-distributed foods, or—in the case of species that defend territories on their wintering grounds—to prospect high-quality territories. The prevalence of these movements may be age-dependent: for example, all individuals may benefit from scouting for ephemeral food resources, while experienced individuals that already hold high-quality territories may be less likely to prospect territories elsewhere on their wintering grounds.

### Exploratory movements on the breeding grounds (juveniles)

Back at the breeding grounds, the annual (cycle) perspective of exploratory movements begins again, but we now focus on juveniles. Although juveniles are inexperienced in terms of breeding, they are not naïve with respect to, e.g., favourable nest sites, and therefore have a reproductive advantage over immigrants to the area lacking the same knowledge [104]. Hatch-year birds may make exploratory movements as soon as they gain independence, moving diurnally to identify habitat to which to disperse [43, 105, 106], to identify potential future breeding grounds [107–111], and/or to identify local landscape features as signposts/landmarks to facilitate returning in subsequent years [108]. However, time constraints may limit the availability of this information. Indeed, it has been argued that prospecting for future breeding areas *within* the active breeding season may be more prevalent in long-distance migrants than short-distance migrants or residents, as they necessarily have less time to initiate migration and therefore less time after the breeding season ends to prospect the area [86]. This constraint affects both juveniles and adults.

Perhaps somewhat surprisingly, then, age-related differences in post-breeding movements have been documented in blackpoll warblers *Setophaga striata*. While the movements of adults in the post-breeding period indicated the commencement of migration, hatch-years instead appear to undertake regional exploratory flights [112]. Similarly, “pronounced non-directional wandering movements” are also documented in juvenile barn swallows *Hirundo rustica* [113] and sand martins *Riparia riparia* [114]. These movements occur well after the breeding season has ended, suggesting they serve a purpose beyond assessing potential future territories. It is argued that birds learn navigational cues, e.g., landmarks, signposts, and/or magnetic information, during their local exploratory flights and thus improve their ability to return to the scouted breeding area during the next spring season. The ultimate benefit is that the bird could make use of prior knowledge gained from the exploratory movements performed there, and thus have higher breeding success than they would if in a novel breeding habitat.

## Pre-migratory flights

The exploratory movements that occur in the transition from the breeding season to migration are extremely understudied in migratory songbirds. While breeding, most avian migrants are stationary, territorial, have relatively low energy stores, and rest during the night. During migration, long-distance migratory songbird are highly mobile, accumulating energy during the day during stopover, and excessive burning excessive energy during nocturnal migratory flights, while sleeping only very little. When transitioning from the stationary breeding period to the mobile migratory period, significant behavioural and physiological changes occur in preparation for migration [52, 53] (Fig. 3). These changes are all the more significant for many songbirds that switch from diurnal activity when breeding to nocturnal activity when migrating, and we therefore hypothesise that pre-migratory flights may more broadly occur within this same transitionary period for long-distance, nocturnally migratory songbirds, though the extent to which an individual, or even a given species, performs such movements remains to be seen.

Speculative evidence for this behaviour comes in the form of nocturnal restlessness, which has been described in a number of long-distance migrants (i.e., [115–117]). Such restlessness has been observed prior to the (migratory) period classified as *Zugunruhe* [116, 118, 119], which may indicate pre-migratory flight behaviour by wild birds held in captivity. Given this background, pre-migratory flights likely have a circadian component, similar to that of *Zugunruhe*. As these observations were not limited to one species, they also suggest that pre-migratory activity is a common behaviour shown by many nocturnal migrant songbirds before departing on migration. Nevertheless, this pre-Zugunruhe restlessness may not be directly related to pre-migratory flights and the link between the two remains to be elucidated, as to date, no study has shown a clear connection between caged birds’ restlessness and pre-migratory flight behaviour.

### Definition of pre-migratory flights

We define pre-migratory flights as nocturnal movements undertaken by a migratory bird during the transitional period between the end of a stationary annual cycle stage and the onset of migration. These flights enable individuals to gain information or experience necessary for migration. In preparation for migration, these birds shift their daily rhythms to nocturnal rhythms and must gain experience with flying at night prior to setting out on migration; we thus speculate these movements are exclusive to nocturnal migrants and must occur at night.

### Current knowledge of pre-migratory flights

To date, the only examples of pre-migratory flights were documented on the Courish Spit in Russia within cohorts of juvenile birds; pre-migratory flights in adults of any species have yet to be studied. Mukhin [54] ringed reed warblers in the nest and subsequently noted their nocturnal post-fledging movements by capturing birds between sunset and sunrise prior to the onset of migration. Twenty-five juveniles between 37 and 50 days of age were caught at night,all were moulting and had low fat scores. Previous work suggests that the youngest age at which juvenile reed warblers initiate migration is approximately 54 days (Mukhin, unpubl. data, [120]). These juveniles were caught 10 km or more away from their natal sites, and predominantly northeast of their natal sites, making it extremely unlikely these juveniles were migrating. Forty other juveniles in a similar age class (mean age = 43 days) were caught during the day, possibly during post-fledging dispersal movements. Given the clear occurrence of diurnal movements, and that diurnally moving birds were not captured moving nocturnally or vice-versa, nocturnal flights likely served a purpose distinct from diurnal flights.

In a follow-up study, Mukhin et al. [55] radio-tracked 27 juvenile reed warblers at their natal site and followed their nocturnal movements. Of those, 16 birds tagged before age 40 d made nocturnal flights that consisted of “flying in different directions over the study area and also disappearing from the reception range [1.5 km], with subsequent reappearance […] the same night”. One bird performed a total of nine pre-migratory flights over two weeks prior to leaving the study area, initiating its flights earlier in the night as the season advanced. These pre-migratory flights nicely illustrate the gradual transition from exclusively diurnal to increasingly nocturnal behaviour.

Despite abundant examples of juvenile migrants making large-scale movements away from their natal territories long before onset of autumn migration [43, 44, 105, 106, 121], further concrete evidence of true pre-migratory flights is lacking. Typically, observations of juvenile relocation prompts speculation that an individual moved to gain access to better food resources [43, 106, 122], avoid predators [123], prospect future breeding sites [124], gain familiarity with landmarks [121], or even to begin migration. We do not dispute any of these hypotheses, but suggest alternatively interpretations that some relocations may reflect side effect of pre-migratory flights. For example, a juvenile moves a noticeable distance (e.g., 1 km within 24 h), and a researcher assumes that between detections on two days, this individual relocated diurnally. However, this bird instead might have performed a nocturnal pre-migratory flight, terminating close to but not directly at its starting location. Unaware of this nocturnal activity, the researcher may incorrectly interpret the purpose of the movement based on a false assumption of when it occurred. Such scenarios will result in pre-migratory flights going undetected, and may be the reason that, to date, true documentation of pre-migratory flights is sparse.

### Hypotheses as to why pre-migratory flights are undertaken

We propose the following hypotheses for pre-migratory flights [55, 112], and apply them to our current knowledge of pre-migratory flights. These hypotheses are not mutually exclusive and not ordered in terms of importance, and we distinguish them from hypotheses pertaining exclusively to post-fledging movements (i.e., in [43]) or ones that address larger-scale diurnal movements (i.e., in [121]).

(i) To practice flying at night
(ii) To practice assessing meteorological conditions aloft
(iii) To assess orientation cues and practice orientation skills
(iv) To create magnetic and/or landscape maps
(v) To practice selecting suitable habitat in darkness

(i) Many long-distance migrant songbirds migrate exclusively during the night [125, 126]. As they forage during the day and live a sheltered life during the night, juveniles have no experience of flying at night. We therefore hypothesise that pre-migratory flights offer the function of practicing nocturnal flights before embarking on migration.
(ii) Migrant birds also assess environmental conditions in the air and time their migratory flights to coincide with favourable conditions aloft (reviewed in [127–130]. It seems plausible that migration-inexperienced juveniles may have to practice their skills to discriminate between favourable and unfavourable environmental conditions. We therefore suggest practicing assessing meteorological conditions aloft as another hypothesis for pre-migratory flights. There is some correlative evidence supporting this hypothesis, because adult, but not juvenile, migrant songbirds avoided headwinds, rain, and low-visibility conditions when initiating autumn migration from the breeding area [131]. That juveniles chose to fly in suboptimal and hence more energetically demanding conditions suggests inexperience regarding how to optimally time their flights to favourable environmental conditions [131] and may explain why juveniles migrate slower with more frequent and longer stopovers than adults [132]. Although quantitative results are currently lacking, we assume that individuals, especially juveniles, practice flying at night prior to the onset of migration and start improving their skills to better assess when favourable meteorological conditions occur. Moreover, we expect that these skills improve over time in juveniles and eventually match those of experienced birds, though we cannot ignore the effect of selective disappearance [133]. Notably, migrants also perform nocturnal exploratory flights at a stopover site during migration hours to days prior to departure [51], perhaps to assess meteorological conditions before deciding whether to depart [127]. Such a flight may also be conducted during the pre-migratory phase, enabling individuals to learn under what conditions movement is optimised. In order to know whether conditions are favourable or unfavourable, a bird requires experience flying in both, which could be explored during pre-migratory flights. However, if a bird either opts not to fly in inclement weather or such weather is not experienced, it may still gain this experience later, while already on migration, and would presumably adjust its tactics after having gained this knowledge.
(iii) To arrive at the wintering grounds, naïve juveniles follow a genetically encoded migration program, which tells birds how long [134] and in which direction [135] to migrate, cf. clock-and-compass orientation [116, 136]. Celestial (stars, sun, skylight polarization pattern) and magnetic (the Earth’s magnetic field) compass cues provide directional information [137, 138]. Although the magnetic compass is innate, the celestial compass needs to be learned. Juveniles do so during ontogeny, probably within about 15–35 days after fledging [139–141]. This overlaps with the period when juveniles have been observed performing pre-migratory flights [54, 55]. Although nocturnal flights are not mandatory to calibrate the magnetic compass relative to the star pattern [139], it would be favourable for juveniles to practice and assess the different compass courses in free-flight and to familiarize themselves with their orientation skills in a known environment before having to rely on those in a novel environment. Nocturnal flights therefore allow for both the development of the compass systems, and the opportunity to practice orienting using these.
(iv) During their first southbound migration, juvenile songbirds are expected to establish magnetic and landscape maps [137], which they use for true navigation during subsequent migrations to find their migratory destination again even if displaced during migration, e.g., [142–144], but see [145]. It is reasonable to assume that juveniles gain experience with the magnetic and landscape features of their natal area prior to leaving it in order to be able return to it in subsequent years, and that they do so at least partly via nocturnal flights. As nocturnal migrants, they therefore must recognize these cues at night. Gaining familiarity with a landscape, and the subsequent formation of a migratory target, has been posited as a primary reason for exploratory movements made by juveniles in reed warblers [54, 55], blackpoll warblers [112, 121, 146], hermit thrushes *Catharus guttatus* [1], and “Ipswich” savannah sparrows *Passerculus sandwichensis princeps* [111]. Likewise, birds “imprint” on their natal site, e.g. on magnetic inclination [147]. Experimental work with collared flycatchers, pied flycatchers *Ficedula hypocleuca*, and juvenile chaffinches *Fringilla coelebs* suggests that when deprived of the ability to explore around the natal area prior to being released at the onset of migration, individuals fail to return in subsequent years [148–150]. Although these studies do not elucidate whether exploration would have occurred in the day or night, it seems probable that exploratory movements at the natal site function to generate or gather cues essential to return in subsequent years.
(v) As many long-distance migratory songbirds migrate exclusively at night [125], they must identify suitable habitats in which to rest, recover, and fuel in between migratory endurance flights prior to sunrise (for a review of habitat selection by nocturnal migrants while on migration, see [151]). In the course of performing exploratory flights around their natal area, birds familiarise themselves with typical cues, as perceived while aloft at night, of favourable habitats (e.g., the currently used site) and novel habitats, which might be less favourable. In doing so, they could gain experience selecting their preferred habitat based on those cues. If migrants assess both favourable and unfavourable feeding habitats prior to beginning migration during pre-migratory flights, this likely improves an individual’s ability to select stopover sites between migratory flights, and therefore increases migration success. For example, habitat cover determines how much body mass can be gained in migrants [152], and as such, the cost of an individual being unable to identify adequate stopover habitat before landing may be the inability to effectively fuel, which in turn prolongs the time until sufficient fuel stores are accumulated for the next migratory flight. Indeed, nocturnal migrants appear to use habitat cover (e.g., extent of hardwood forest), as an indicator of habitat quality which directly informs the decision to end a migratory flight during the night [153]. As migrants use stopovers to maximise their direct or indirect fitness [154], the quality of the habitat they choose at which to stop over is critical, and the ability to select such habitat at night is therefore paramount.

*Addressing movements that may be mistakenly identified as pre-migratory flights (“false positives”)*

If the flight that would initiate autumn migration is aborted, it may be erroneously classified as a pre-migratory flight. However, as flights that begin or resume migration usually occur shortly after sunset in nocturnal migrants [126, 155], most flights that are initiated later in the night are unlikely to be departure events. Similarly, nocturnal flights to escape a predator or in response to human-caused disturbances, such as fireworks [156] and helicopters [157], could potentially be misinterpreted as pre-migratory flights. Nevertheless, given clear evidence of birds performing nocturnal flights absent such disturbances [55] and prior to reaching a migratory state [54], it is plausible that most pre-migratory, nocturnal movements are indeed pre-migratory flights.

### Extent of pre-migratory flights

**Fig. 2:**
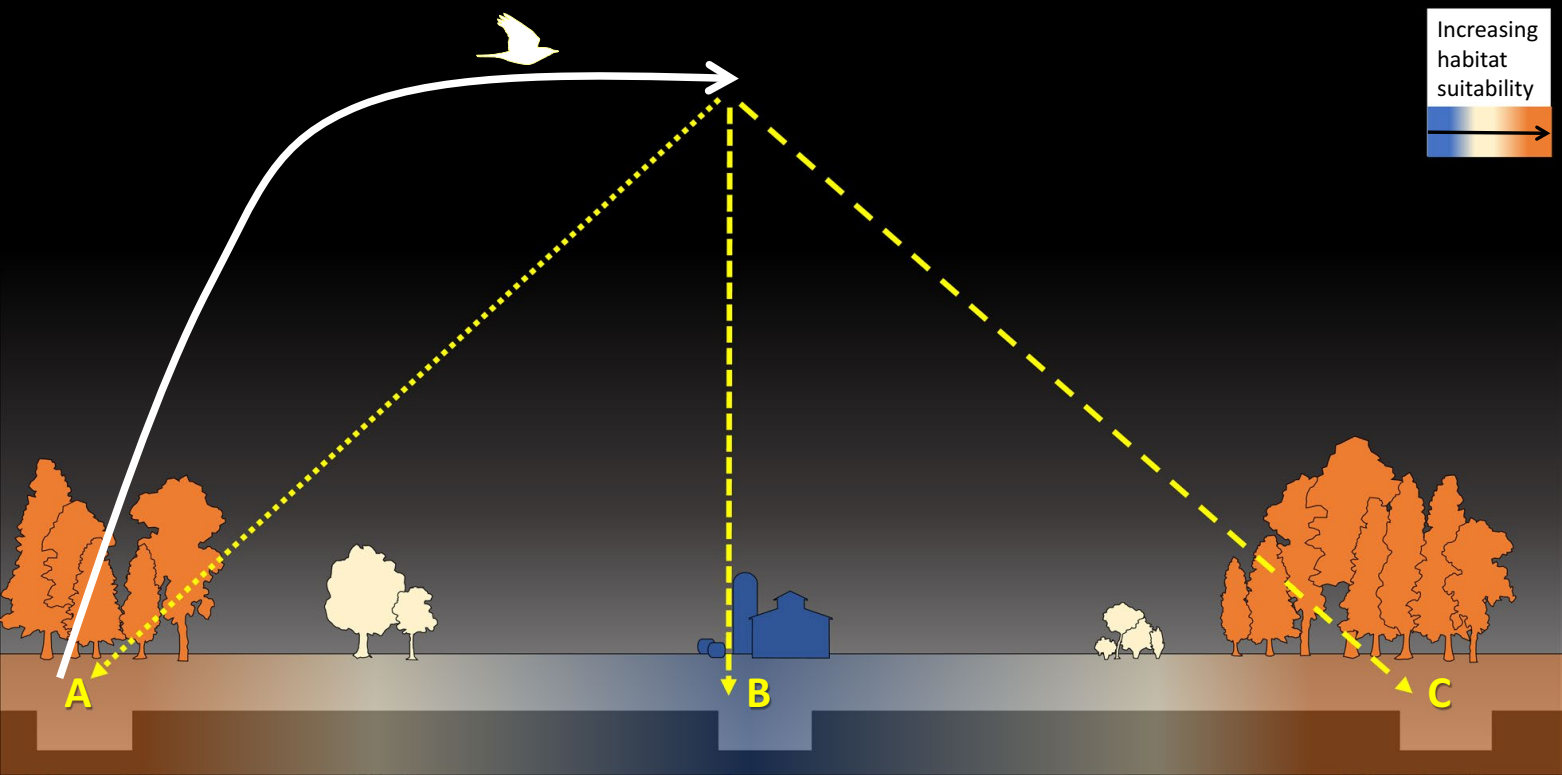
Different extents of pre-migratory flights might serve different functions. For the case of our model, a woodland thrush, suitable habitat is shown in orange, and unsuitable habitat is shown in blue. The bird takes off from its starting point (solid white line) and the extent of its movement depends on the function of the flight. **A** Small-scale flights serve to practice flying at night, assessing meteorological conditions, or are a function of avoiding a perceived threat and likely terminate at or near the starting location. **B** Intermediate- and large-scale movements serve to practice and assess orientation skills and form landscape or magnetic maps; the scale to which they occur may depend on the surrounding landscape or the existing status of an individual’s internal maps. The scale of flights that serve to practice selecting suitable habitat in darkness may depend on the extent to which surrounding habitat is contiguous. For illustrative purposes, we show these larger-scale flights terminating far from the starting point, though an individual may instead circle back. Although the intermediate flight, B, shows the bird landing in unsuitable habitat, this does not suggest that all flights on this scale terminate in unfavourable landscapes, merely that landing in unsuitable habitat is a possible outcome in the context of pre-migratory flights

**Fig. 3:**
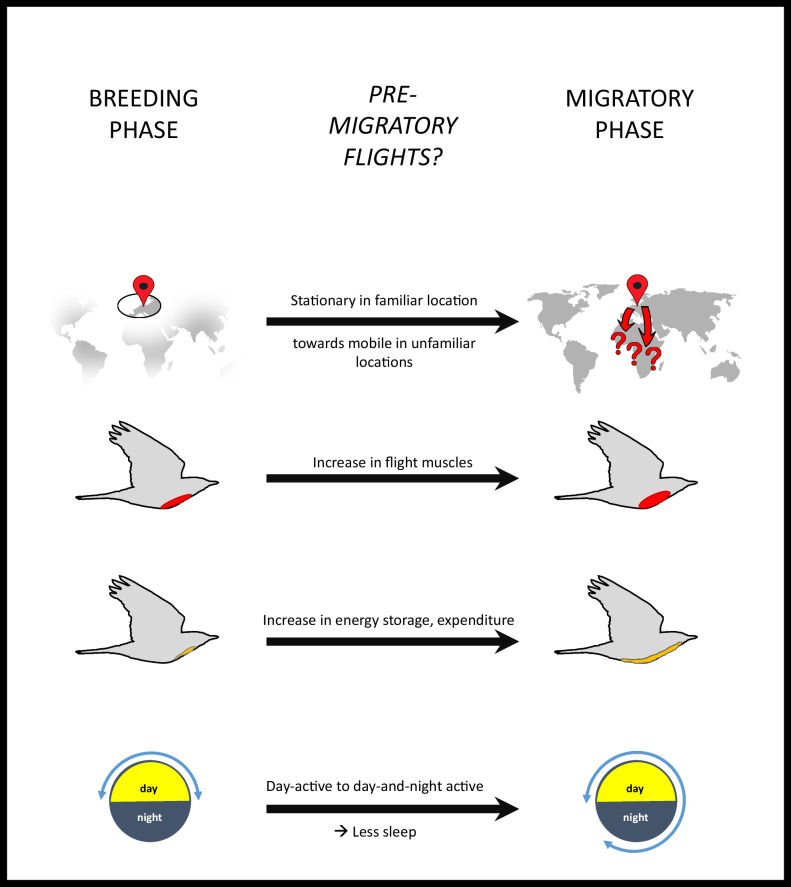
Physiological changes during the transitional period between breeding and migration

Although we expect these hypotheses to operate in tandem, we argue that the extent and duration to fulfil the specific functions of a pre-migratory flight differ (Table 1). We expect that flights that serve to assess conditions and make first forays into nocturnal activity are relatively short, as too would flights made in response to a perceived threat be, e.g. on the scale of minutes. Flights whose purpose is to create maps and practice orientation are likely longer, perhaps on the scale of hours, and must occur on a much larger scale, e.g. several kilometres (Fig. 2). For habitat selection, the extent to which surrounding habitat is contiguous must play a role in how far a bird must fly to be able to distinguish between favourable and unfavourable places to land. It remains speculative whether birds necessarily return to the initial point of departure or land elsewhere in the broader natal/breeding area (Figs. 2 and 3). For one, depending on an individual’s broader familiarity with its starting location, it may land elsewhere from its starting point yet in an area still familiar to it. Furthermore, the scale of movement itself is likely species- and landscape-dependent, so a “small-scale” pre-migratory flight for one species may constitute a “large-scale” pre-migratory flight for another. To this end, we hesitate to speculate about the exact spatio-temporal extent of pre-migratory flights that we may observe, as the scope of these flights may be highly dependent on other intrinsic and extrinsic factors, which we discuss below.

**Table 1:**
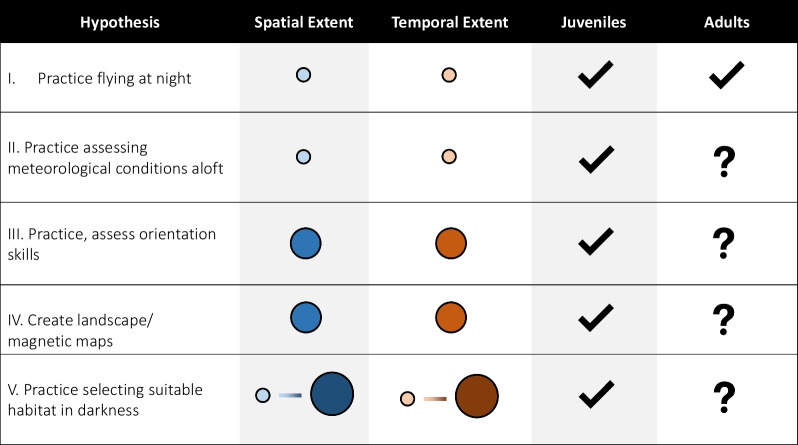
Speculated hypothesis-specific spatio- (blue) and temporal- (orange) extents of pre-migratory flights

The size of the circles represent the speculated extent (small, large, or a range) of the flights necessary to fulfil the stated purpose of the movement. For example, simply practicing nocturnal flights would not require large flights of a long duration, while creating maps would require longer time aloft over more area

The extent to which pre-migratory flights occur within a population is unclear, and is probably subject to intrinsic and extrinsic factors. For example, recently fledged, weak, injured, undernourished, or otherwise stressed individuals are unlikely to make pre-migratory flights, independent of when they depart on migration. Instead, only individuals capable of flight and in good body condition likely carry out these movements. Likewise, although we anticipate that inexperienced juveniles may conduct pre-migratory flights in unfavourable weather, it is also possible that individuals terminate pre-migratory flight attempts in such conditions, or opt not to embark at all. In contrast, good visibility of celestial cues, i.e., clear conditions, may instead trigger individuals to engage in these flights.

To date, as only juveniles have been studied within this context, the only evidence of pre-migratory flights is also currently limited to juveniles. While many of our hypotheses support the idea that pre-migratory flights allow juveniles to gain experience prior to departing on their first migration, we think it plausible that adults also embark on nocturnal movements before migration as they undergo the physiological and behavioural shifts from breeding to a state of migratory preparation. As migration in general is endogenously controlled [116, 136], we assume the same for pre-migratory flights, and as Brown and Taylor noted [158], “perhaps when individuals are in a migratory state they simply have an instinctive urge to fly”.

### Biological significance of pre-migratory flights

A full life-cycle approach will aid in our understanding of the life history of avian migrants [159, 160]. Such an approach includes assessing the consequences of migration, a period during which migrants experience the highest mortality rates within their annual cycle [161–165].

We speculate that individuals that perform pre-migratory flights may have the experience necessary to contend with challenges that might otherwise negatively affect their migration timings, i.e., knowledge of how to respond to differing weather conditions or how to quickly course-correct if blown off course. These timings are noteworthy as they may have cascading effects throughout an individual’s life in the form of indirect fitness consequences. Individuals that arrive early to their wintering grounds may competitively exclude other individuals from high-quality habitat [166], and may settle first in lands where their return migration is shorter, setting themselves up for early returns to their breeding grounds [167]. Individuals that arrive earlier to their breeding grounds may reap the benefits of pairing earlier, laying larger clutches, and fledging more young than later-arriving individuals [168, 169]. Taken together, optimal arrival timing at migratory destinations will result in delayed, albeit significant, fitness benefits (i.e., [93], but see also [170, 171]).

We postulate that individuals performing pre-migratory flights have higher survival on migration, return earlier to their breeding grounds, and may ultimately have higher reproductive success over conspecifics that do not perform pre-migratory flights. Furthermore, flying is probably not as energetically costly as commonly assumed. Depending on conditions, it may be even less demanding to fly than to simply stay put and try to keep warm [172], and indeed, previously described large-scale, exploratory flights suggest that they are not costly [158]. We therefore assume that the fitness benefits conferred by pre-migratory flights outweigh their costs, and expect pre-migratory flights to be widespread among long-distance migrants.

## Conclusions

Pre-migratory flights are a rarely detected form of exploratory movement that likely have an important biological significance. These flights serve as a means by which individuals can gain experience relevant to migration before departure, and individuals that perform them likely experience higher immediate and delayed fitness benefits than those that do not. Despite their assumed significant role for long-distance migratory songbirds, we know next to nothing about their prevalence among nocturnal migrants, and their spatiotemporal extent. We urge researchers to be aware of the potential for these flights, and to design studies in which they can be detected and interpreted within the framework of migratory movement.

## Data Availability

Not applicable.

## References

[1] Berrigan LE. Post-breeding movements of Swainson's Thrush in Southern Nova Scotia [Master's thesis, Acadia University]. 2018.

[2] Collett TS, Zeil J (2018). Insect learning flights and walks. Curr Biol.

[3] Degen J (2016). Honeybees learn landscape features during exploratory orientation flights. Curr Biol.

[4] Zeil J, Fleischmann PN (2019). The learning walks of ants (Hymenoptera: Formicidae). Myrmecol News.

[5] Boyd DK, Pletscher DH (1999). Characteristics of dispersal in a colonizing wolf population in the central rocky mountains. J Wildl Manag.

[6] Boydston EE (2005). Sexually dimorphic patterns of space use throughout ontogeny in the spotted hyena (Crocuta crocuta). J Zool.

[7] Dahl F, Willebrand T (2005). Natal dispersal, adult home ranges and site fidelity of mountain hares Lepus timidus in the boreal forest of Sweden. Wildlife Biol.

[8] Garrett MG, Franklin WL (1988). Behavioral ecology of dispersal in the black-tailed prairie dog. J Mammal.

[9] Höner OP (2007). Female mate-choice drives the evolution of male-biased dispersal in a social mammal. Nature.

[10] Morrison CD, Boyce MS, Nielsen SE (2015). Space-use, movement and dispersal of sub-adult cougars in a geographically isolated population. PeerJ.

[11] Shrader AM, Owen-Smith N (2002). The role of companionship in the dispersal of white rhinoceroses (Ceratotherium simum). Behav Ecol Sociobiol.

[12] Simcharoen A et al. Exploratory dispersal movements by young tigers in Thailand’s western forest complex: the challenges of securing a territory. Mammal Res. 2021.

[13] Van Deelen TR. Dispersal patterns of juvenile beavers in western Montana. 1991.

[14] Debeffe L (2013). Exploration as a key component of natal dispersal: dispersers explore more than philopatric individuals in roe deer. Anim Behav.

[15] Debeffe L (2014). A one night stand? Reproductive excursions of female roe deer as a breeding dispersal tactic. Oecologia.

[16] Doolan SP, Macdonald DW (1996). Dispersal and extra-territorial prospecting by slender-tailed meerkats (Suricata suricatta) in the south-western Kalahari. J Zool.

[17] Höner OP (2005). The effect of prey abundance and foraging tactics on the population dynamics of a social, territorial carnivore, the spotted hyena. Oikos.

[18] Lovari S (2008). Going out to mate: excursion behaviour of female roe deer. Ethology.

[19] Salsbury CM, Armitage KB (1994). Home-range size and exploratory excursions of adult, male yellow-bellied marmots. J Mammal.

[20] Joly P. Behavior in a changing landscape: using movement ecology to inform the conservation of pond-breeding amphibians. Front Ecol Evolut. 2019;7(155).

[21] Gruber J (2017). Geographic divergence in dispersal-related behaviour in cane toads from range-front versus range-core populations in Australia. Behav Ecol Sociobiol.

[22] Miller AJ, Page RA, Bernal XE (2018). Exploratory behavior of a native anuran species with high invasive potential. Anim Cogn.

[23] Tsellarius AY, Tsellarius EY (2003). An access to the females as a resource of male’s territory in Lacerta saxicola. Herpetologia Petropolitana.. Proc 12th Ord Gen Meet Soc Eur Herpetol.

[24] Smithson EB, Johnston CE (1999). Movement patterns of stream fishes in a Ouachita highlands stream: an examination of the restricted movement paradigm. Trans Am Fish Soc.

[25] Brightsmith DJ et al. Satellite telemetry reveals complex migratory movement patterns of two large macaw species in the western Amazon basin. Avian Conserv Ecol 2021;16(1).

[26] Gass CL, Montgomerie RD. Hummingbird foraging behavior: decision-making and energy regulation. Foraging behavior: ecological, ethological, and psychological approaches. 1981;159–194.

[27] Hutto RL, Cody ML (1985). Habitat selection by nonbreeding, migratory land birds. Habitat selection in birds.

[28] Smetzer JR, Paxton KL, Paxton EH (2021). Individual and seasonal variation in the movement behavior of two tropical nectarivorous birds. Mov Ecol.

[29] Ausprey IJ, Rodewald AD (2013). Post-fledging dispersal timing and natal range size of two songbird species in an urbanizing landscape : sincronización de la dispersión posterior al emplumamiento y del tamaño del rango natal de dos especies de aves canoras en un paisaje urbanizado. Condor.

[30] Krüger S, Amar A (2017). Insights into post-fledging dispersal of Bearded Vultures Gypaetus barbatus in southern Africa from GPS satellite telemetry. Bird Study.

[31] Ramos RF (2019). Spatial behaviour of Spanish imperial eagle Aquila adalberti juveniles during the dependence period revealed by high-resolution GPS tracking data. J Ornithol.

[32] Campioni L, Granadeiro JP, Catry P (2017). Albatrosses prospect before choosing a home: intrinsic and extrinsic sources of variability in visit rates. Anim Behav.

[33] Ciaglo M (2021). Evidence of postbreeding prospecting in a long-distance migrant. Ecol Evol.

[34] Todd L et al. Pre-migratory movements by juvenile burrowing owls in a patchy landscape. Avian Conserv Ecol 2007;2(2).

[35] Wolfson DW, Fieberg JR, Andersen DE (2020). Juvenile Sandhill Cranes exhibit wider ranging and more exploratory movements than adults during the breeding season. Ibis.

[36] Earl JE (2016). Characteristics of lesser prairie-chicken (*Tympanuchus pallidicinctus*) long-distance movements across their distribution. Ecosphere.

[37] Vogel JA, Shepherd SE, Debinksi DM (2015). An unexpected journey: greater prairie-chicken travels nearly 4000 km after translocation to Iowa. Am Midland Natural.

[38] Burstal J (2020). Radiotracking invasive spread: are common mynas more active and exploratory on the invasion front?. Biol Invasions.

[39] Smetzer JR (2021). Automated telemetry reveals post-reintroduction exploratory behavior and movement patterns of an endangered corvid, ʻAlalā (Corvus hawaiiensis) in Hawaiʻi, USA. Global Ecol Conserv.

[40] Tobler M, Smith HG (2004). Specific floater home ranges and prospective behaviour in the European starling. Sturnus vulgaris Naturwissenschaften.

[41] Mayer M, Zedrosser A, Rosell F (2017). Extra-territorial movements differ between territory holders and subordinates in a large, monogamous rodent. Sci Rep.

[42] Cox AS, Kesler DC (2012). Prospecting behavior and the influence of forest cover on natal dispersal in a resident bird. Behav Ecol.

[43] Vega Rivera JH, Rappole JH, McShea WJ, Haas CA (1998). Wood thrush postfledging movements and habitat use in Northern Virginia. Condor.

[44] White JD, Faaborg J. Post-fledging movement and spatial habitat-use patterns of juvenile swainson’s thrushes. Wilson J Ornithol. 2008;120(1):62–73.

[45] Aubry KB, Koehler GM, Squires JR. Ecology of Canada lynx in southern boreal forests [Chapter 13]. In: Ruggiero LF, Aubry KB, Buskirk SW, Koehler GM, Krebs, CJ, McKelvey, KS, Squires JR editor Ecology and conservation of lynx in the United States. Gen. Tech. Rep. RMRS-GTR-30WWW. Fort Collins, CO: US Department of Agriculture, Forest Service, Rocky Mountain Research Station. 2000; p. 373–396. 30:373–396.

[46] Arlt D, Pärt T (2008). Post-breeding information gathering and breeding territory shifts in northern wheatears. J Anim Ecol.

[47] Reed JM et al. Informed Dispersal. In Nolan V, Ketterson ED, Thompson CF editors Current Ornithol. pp 189–259. Boston: Springer; 1999.

[48] Doerr ED, Doerr VAJ (2005). Dispersal range analysis: quantifying individual variation in dispersal behaviour. Oecologia.

[49] Kesler DC, Walters JR, Kappes JJ (2010). Social influences on dispersal and the fat-tailed dispersal distribution in red-cockaded woodpeckers. Behav Ecol.

[50] Soulsbury CD (2011). Behavioral and spatial analysis of extraterritorial movements in red foxes (Vulpes vulpes). J Mammal.

[51] Schmaljohann H (2011). Nocturnal exploratory flights, departure time, and direction in a migratory songbird. J Ornithol.

[52] Ramenofsky M, Wingfield JC (2017). Regulation of complex behavioural transitions: migration to breeding. Anim Behav.

[53] Ramenofsky M, Wingfield JC (2006). Behavioural and physiological conflicts in migrants: the transition between migration and breeding. J Ornithol.

[54] Mukhin A (2004). Night movements of young reed warblers (*Acrocephalus scirpaceus*) in summer: is it postfledging dispersal?. Auk.

[55] Mukhin A, Kosarev V, Ktitorov P (2005). Nocturnal life of young songbirds well before migration. Proc R Soc London B.

[56] Mettke-Hoffman C, Gwinner E (2004). Differential assessment of environmental information in a migratory and a nonmigratory passerine. Anim Behav.

[57] Howlett JS, Stutchbury BJM (2003). Determinants of between-season site, territory, and mate fidelity in hooded warblers (Wilsonia Citrina). Auk.

[58] Lawn MR (1982). Pairing systems and site tenacity of the willow warbler phylloscopus trochilus in southern England. Ornis Scand.

[59] Bensch S, Hasselquist D (1991). Territory infidelity in the polygynous great reed warbler Acrocephalus arundinaceus: the effect of variation in territory attractiveness. J Anim Ecol.

[60] Jaakkonen T, Kari A, Forsman JT (2013). Flycatchers copy conspecifics in nest-site selection but neither personal experience nor frequency of tutors have an effect. PLoS ONE.

[61] Morinay J (2020). Behavioural traits modulate the use of heterospecific social information for nest site selection: experimental evidence from a wild bird population. Proc R Soc B.

[62] Samplonius JM, Both C (2017). Competitor phenology as a social cue in breeding site selection. J Anim Ecol.

[63] Szymkowiak J, Thomson RL, Kuczyński L (2016). Wood warblers copy settlement decisions of poor quality conspecifics: support for the tradeoff between the benefit of social information use and competition avoidance. Oikos.

[64] Szymkowiak J, Thomson RL, Kuczyński L (2017). Interspecific social information use in habitat selection decisions among migrant songbirds. Behav Ecol.

[65] Hahn BA, Silverman ED (2006). Social cues facilitate habitat selection: American redstarts establish breeding territories in response to song. Biol Let.

[66] Rushing CS, Dudash MR, Marra PP (2015). Habitat features and long-distance dispersal modify the use of social information by a long-distance migratory bird. J Anim Ecol.

[67] Ward MP, Schlossberg S (2004). Conspecific attraction and the conservation of territorial songbirds. Conserv Biol.

[68] Kelly JK, Ward MP (2017). Do songbirds attend to song categories when selecting breeding habitat? A case study with a wood warbler. Behaviour.

[69] Amrhein V, Kunc HP, Naguib M (2004). Non-territorial nightingales prospect territories during the dawn chorus. Proc R Soc London B Biol Sci..

[70] Dale S (1990). Mate sampling behaviour of female pied flycatchers: evidence for active mate choice. Behav Ecol Sociobiol.

[71] Roth T (2009). Sex-specific timing of mate searching and territory prospecting in the nightingale: nocturnal life of females. Proc R Soc B Biol Sci.

[72] Bensch S, Hasselquist D (1992). Evidence for active female choice in a polygynous warbler. Anim Behav.

[73] Alatalo RV, Lundberg A, Glynn C (1986). Female pied flycatchers choose territory quality and not male characteristics. Nature.

[74] Byers BE (2004). Extrapair paternity increases variability in male reproductive success in the chestnut-sided warbler (Dendroica Pensylvanica), socially monogamous songbird. Auk.

[75] Canal D, Jovani R, Potti J (2012). Male decisions or female accessibility? Spatiotemporal patterns of extra pair paternity in a songbird. Behav Ecol.

[76] Churchill JL, Hannon SJ (2009). Off-territory movement of male American Redstarts (Setophaga ruticilla) in a fragmented agricultural landscape is related to song rate, mating status and access to females. J Ornithol.

[77] Pedersen MC, Dunn PO, Whittingham LA (2006). Extraterritorial forays are related to a male ornamental trait in the common yellowthroat. Anim Behav.

[78] Reitsma LR (2018). Extra-pair paternity in a long-distance migratory songbird beyond neighbors’ borders and across male age classes. Can J Zool.

[79] Stapleton MK, Robertson RJ (2006). Female tree swallow home-range movements during their fertile period as revealed by radio-tracking. Wilson J Ornithol.

[80] Woolfenden BE, Stutchbury BJM, Morton ES (2005). Male acadian flycatchers, empidonax virescens, obtain extrapair fertilizations with distant females. Anim Behav.

[81] Norris DR, Stutchbury BJM (2001). Extraterritorial movements of a forest songbird in a fragmented landscape. Conserv Biol.

[82] Dolan AC (2007). Extrapair paternity and the opportunity for sexual selection in a socially monogamous passerine. Behav Ecol.

[83] Kaiser SA (2017). Ecological and social factors constrain spatial and temporal opportunities for mating in a migratory songbird. Am Nat.

[84] Whitaker DM, Warkentin IC (2010). Spatial ecology of migratory passerines on temperate and boreal forest breeding grounds. Auk.

[85] Stutchbury BJ, Robertson RJ (1987). Behavioral tactics of subadult female floaters in the tree swallow. Behav Ecol Sociobiol.

[86] Dale S (2006). How do birds search for breeding areas at the landscape level? Interpatch movements of male ortolan buntings. Ecography.

[87] Doligez B, Pärt T, Danchin E (2004). Prospecting in the collared flycatcher: gathering public information for future breeding habitat selection?. Anim Behav.

[88] Ward MP (2005). Habitat selection by dispersing yellow-headed blackbirds: evidence of prospecting and the use of public information. Oecologia.

[89] Cooper NW, Marra PP (2020). Hidden long-distance movements by a migratory bird. Curr Biol.

[90] Bäckman J, Andersson A, Pedersen L, Sjöberg S, Tøttrup AP, Alerstam T (2017). Actogram analysis of free-flying migratory birds: new perspectives based on acceleration logging. J Comp Physiol A.

[91] Smith MG (2020). Extraterritorial visits in a cooperatively breeding songbird are consistent with multiple functions. Anim Behav.

[92] Aebischer A, et al. The role of territory choice, mate choice and arrival date on breeding success in the Savi’s Warbler locustella luscinioides. J Avian Biol. 1996;27(2):143–52.

[93] Lozano GA, Perreault S, Lemon RE (1996). Age, arrival date and reproductive success of male American redstarts Setophaga ruticilla. J Avian Biol.

[94] Tomotani BM (2017). Early arrival is not associated with more extra-pair fertilizations in a long-distance migratory bird. J Avian Biol.

[95] Low M (2015). Delayed timing of breeding as a cost of reproduction. J Avian Biol.

[96] Mills AM (2011). Passerines use nocturnal flights for landscape-scale movements during migration stopover. Condor.

[97] Paxton KL, Van Riper CIII, O’Brien C. Movement patterns and stopover ecology of Wilson’s Warblers during spring migration on the lower Colorado River in Southwestern Arizona. Condor. 2008;110(4):672–81.

[98] Seewagen CL, Slayton EJ, Guglielmo CG (2010). Passerine migrant stopover duration and spatial behaviour at an urban stopover site. Acta Oecologica.

[99] Taylor PD (2011). Landscape movements of migratory birds and bats reveal an expanded scale of stopover. PlosOne.

[100] Schmaljohann H, Eikenaar C (2017). How do energy stores and changes in these affect departure decisions by migratory birds?—a critical view on stopover ecology studies and some future perspective. J Comp Physiol A.

[101] Aborn DA, Moore FR (1997). Pattern of movement by summer Tanagers (*Piranga rubra*) during migratory stopover: a telemetry study. Behaviour.

[102] Moore FR, Aborn DA (2000). Mechanisms of en route habitat selection: How do migrants make habitat decisions during stopover?. Stud Avian Biol.

[103] Gómez C (2017). Fuel loads acquired at a stopover site influence the pace of intercontinental migration in a boreal songbird. Sci Rep.

[104] Bensch S (1998). Higher fitness for philopatric than for immigrant males in a semi-isolated population of great reed warblers. Evolution.

[105] Anders AD, Faaborg J, Thompson FR (1998). Postfledging dispersal, habitat use, and home-range size of juvenile wood thrushes. Auk.

[106] Sim IMW, Ludwig SC, Grant MC, Loughrey JL, Rebecca GW, Reid JM (2013). Postfledging survival, movements, and dispersal of ring ouzels (Turdus torquatus). Auk.

[107] Betts MG (2008). Social information trumps vegetation structure in breeding-site selection by a migrant songbird. Proc R Soc B Biol Sci.

[108] Nielsen B, Bensch S (1995). Post-fledging movements of juvenile Reed Warblers Acrocephalus scirpaceus and Sedge Warblers Acrocephalus schoenobaenus. Ornis Svecica.

[109] Nocera JJ, Forbes GJ, Giraldeau L-A (2006). Inadvertent social information in breeding site selection of natal dispersing birds. Proc R Soc B Biol Sci.

[110] Pegan TM (2018). Solar-powered radio tags reveal patterns of post-fledging site visitation in adult and juvenile tree swallows tachycineta bicolor. PLoS ONE.

[111] Crysler Z. Breeding ground dispersal and fall migratory movements of Ipswich Sparrows (*Passerculus sandwichensis princeps*), Acadia University. 2015.

[112] Brown JM, Taylor PD (2015). Adult and hatch-year blackpoll warblers exhibit radically different regional-scale movements during post-fledging dispersal. Biol Let.

[113] Ormerod SJ (1991). Pre-migratory and migratory movements of Swallows Hirundo rustica in Britain and Ireland. Bird Study.

[114] Mead CJ, Harrison JD (1979). Sand Martin movements within Britain and Ireland. Bird Study.

[115] Bulte M, Bairlein F (2013). Endogenous control of migratory behavior in Alaskan Northern Wheatears *Oenanthe oenanthe*. J Ornithol.

[116] Gwinner E (1996). Circadian and circannual programmes in avian migration. J Exp Biol.

[117] Mukhin A (1999). Nocturnal restlessness in caged jevenile Reed Warblers *Acrocephalus scirpaceus*. Avian Ecol Behav.

[118] Gwinner E, Schwabl-Benzinger I. Adaptive temporal programming of molt and migratory disposition in two closely related long-distance migrants, the pied flycatcher (Ficedula hypoleuca) and the collared flycatcher (Ficedula albicollis). In Avian navigation, pp. 75–89. Springer; 1982.

[119] Van Doren BM, Liedvogel M, Helm B (2017). Programmed and flexible: long-term Zugunruhe data highlight the many axes of variation in avian migratory behaviour. J Avian Biol.

[120] Ölschlegel H (1990). Über das Ortsverhalten junger Teichrohrsänger, *Acrocephalus scirpaceus*, nach dem Selbständigwerden. Beiträge zur Vogelkunde.

[121] Mitchell GW, Taylor PD, Warkentin IG (2010). Assessing the function of broad-scale movements made by juvenile songbirds prior to migration. Condor Ornithol Appl.

[122] Morton ML, Wakamatsu MW, Pereyra ME, Morton GA. Postfledging dispersal, habitat imprinting, and philopatry in a montane, migratory sparrow. Ornis Scandinavica. 1991;98–106.

[123] Jenkins JMA, Thompson FR, Faaborg J (2017). Behavioral development and habitat structure affect postfledging movements of songbirds. J Wildl Manag.

[124] Styles P, Patchett R, King JR, Cresswell W (2021). Movements of cyprus wheatear *Oenanthe cypriaca* fledglings: evidence of a post-fledging home range away from the natal site prior to first migration. J Ornithol.

[125] Dorka V (1966). Das jahres- und tageszeitliche Zugmuster von Kurz- und Langstreckenziehern nach Beobachtungen auf den Alpenpässen Cou/Bretolet (Wallis). Ornithol Beob.

[126] Müller F (2016). Towards a conceptual framework for explaining variation in the nocturnal departure time of songbird migrants. Mov Ecol.

[127] Liechti F. Birds: blowin’ by the wind? J Ornithol. 2006;147(2):202–11.

[128] Richardson WJ (1978). Timing and amount of bird migration in relation to weather: a review. Oikos.

[129] Richardson WJ (1990). Wind and orientation of migrating birds: a review. Experientia.

[130] Shamoun-Baranes J, Liechti F, Vansteelant WMG (2017). Atmospheric conditions create freeways, detours and tailbacks for migrating birds. J Comp Physiol A.

[131] Mitchell GW (2012). Timing of breeding carries over to influence migratory departure in a songbird: an automated radiotracking study. J Anim Ecol.

[132] Crysler ZJ, Ronconi RA, Taylor PD (2016). Differential fall migratory routes of adult and juvenile Ipswich Sparrows (Passerculus sandwichensis princeps). Mov Ecol.

[133] Sergio F (2014). Individual improvements and selective mortality shape lifelong migratory performance. Nature.

[134] Berthold P, Querner U (1981). Genetic basis of migratory behavior in European warblers. Science.

[135] Helbig AJ (1991). Inheritance of migratory direction in a bird species: a cross-breeding experiment with SE- and SW-migrating blackcaps (Sylvia atricapilla). Behav Ecol Sociobiol.

[136] Berthold P (2001). Bird migration: a general survey.

[137] Mouritsen H (2018). Long-distance navigation and magnetoreception in migratory animals. Nature.

[138] Wiltschko W, Wiltschko R. Magnetic orientation and celestial cues in migratory orientation. In: Berthold P, editor Orientation in birds. Basel: Birkhäuser. 1991. 10.1007/978-3-0348-7208-9_2 1838513

[139] Michalik A (2014). Star compass learning: how long does it take?. J Ornithol.

[140] Weindler P, Baumetz M, Wiltschko W (1997). The direction of celestial rotation influences the development of stellar orientation in young Garden warblers (Sylvia borin). J Exp Biol.

[141] Wiltschko W (1987). The development of the star compass in Garden Warbler, Sylvia borin. Ethology.

[142] Kishkinev D (2021). Navigation by extrapolation of geomagnetic cues in a migratory songbird. Curr Biol.

[143] Perdeck AC. Two types of orientation in migrating *Sturnus vulgaris* and *Fringilla coelebs* as revealed by displacement experiments. Ardea 1958;133–139.

[144] Thorup K (2007). Evidence for a navigational map stretching across the continental U.S. in a migratory songbird. Proc Natl Acad Sci.

[145] Thorup K (2020). Flying on their own wings: young and adult cuckoos respond similarly to long-distance displacement during migration. Sci Rep.

[146] Cormier DA, Taylor PD. Contrasting patterns of post-fledging movements of two sympatric warbler species with different life-history strategies. J Avian Biol. 2019;50(12).

[147] Wynn J (2020). Natal imprinting to the Earth’s magnetic field in a pelagic seabird. Curr Biol.

[148] Berndt R, Winkel W (1979). Verfrachtungs-Experimente zur Frage der Geburtsortsprägung beim Trauerschnäpper (Ficedula hypoleuca). J Ornithol.

[149] Löhrl H (1959). Zur Frage des Zeitpunktes einer Prägung auf die Heimatregion beim Halsbandschnäpper (*Ficedula albicollis*). J Ornithol.

[150] Sokolov LV (1984). The testing of the ability for imprinting and finding the site of future nesting in young Chaffinches. Zoologichesky Zhurnal.

[151] Chernetsov N. Habitat selection by nocturnal passerine migrants en route: mechanisms and results. J Ornithol. 2006;1–7.

[152] Ktitorov P, Bairlein F, Dubinin M (2008). The importance of landscape context for songbirds on migration: body mass gain is related to habitat cover. Landscape Ecol.

[153] Buler JJ, Moore FR, Woltmann S (2007). A multi-scale examination of stopover habitat use by birds. Ecology.

[154] Schmaljohann H, Eikenaar C, Sapir N (2022). Understanding the ecological and evolutionary function of stopover in migrating birds. Biol Rev.

[155] Müller F (2018). Nocturnal departure timing in songbirds facing distinct migratory challenges. J Anim Ecol.

[156] Shamoun-Baranes J (2011). Birds flee en mass from New Year’s Eve fireworks. Behav Ecol.

[157] Mott DF. Influence of low-flying helicopters on the roosting behavior of blackbirds and starlings. 1983.

[158] Brown JM, Taylor PD (2017). Migratory blackpoll warblers (Setophaga striata) make regional-scale movements that are not oriented toward their migratory goal during fall. Mov Ecol.

[159] Holmes RT (2007). Understanding population change in migratory songbirds: long-term and experimental studies of neotropical migrants in breeding and wintering areas. Ibis.

[160] Marra PP (2015). A call for full annual cycle research in animal ecology. Biol Let.

[161] Buechley ER (2021). Differential survival throughout the full annual cycle of a migratory bird presents a life-history trade-off. J Anim Ecol.

[162] Klaassen RHG (2014). When and where does mortality occur in migratory birds? Direct evidence from long-term satellite tracking of raptors. J Anim Ecol.

[163] Loonstra AHJ (2019). Adverse wind conditions during northward Sahara crossings increase the in-flight mortality of black-tailed godwits. Ecol Lett.

[164] Sergio F (2019). When and where mortality occurs throughout the annual cycle changes with age in a migratory bird: individual vs population implications. Sci Rep.

[165] Sillett TS, Holmes RT (2002). Variation in survivorship of a migratory songbird throughout its annual cycle. J Anim Ecol.

[166] Marra PP, Hobson KA, Holmes RT (1998). Linking winter and summer events in a migratory bird by using stable-carbon isotopes. Science.

[167] Studds CE, Kyser TK, Marra PP (2008). Natal dispersal driven by environmental conditions interacting across the annual cycle of a migratory songbird. Proc Natl Acad Sci.

[168] Kokko H (1999). Competition for early arrival in birds. J Anim Ecol.

[169] Potti J, Montalvo S (1991). Male arrival and female mate choice in pied flycatchers ficedula hypoleuca in central Spain. Ornis Scand.

[170] Briedis M (2018). Linking events throughout the annual cycle in a migratory bird—non-breeding period buffers accumulation of carry-over effects. Behav Ecol Sociobiol.

[171] Gow EA (2019). A range-wide domino effect and resetting of the annual cycle in a migratory songbird. Proc R Soc B Biol Sci.

[172] Wikelski M, Tarlow EM, Raim A, Diehl RH, Larkin RP, Visser GH (2003). Costs of migration in free-flying songbirds. Nature.

